# Nanosecond electric pulses are equally effective in electrochemotherapy with cisplatin as microsecond pulses

**DOI:** 10.2478/raon-2023-0049

**Published:** 2023-11-30

**Authors:** Angelika Vizintin, Stefan Markovic, Janez Scancar, Jerneja Kladnik, Iztok Turel, Damijan Miklavcic

**Affiliations:** Faculty of Electrical Engineering, University of Ljubljana, Ljubljana, Slovenia; Department of Environmental Sciences, Jožef Stefan Institute, Ljubljana, Slovenia; Faculty of Chemistry and Chemical Technology, University of Ljubljana, Ljubljana, Slovenia

In Figure 3A, three horizontal bars representing the standard deviation were incorrectly drawn. The corrected figure is shown below.

**FIGURE 3.**
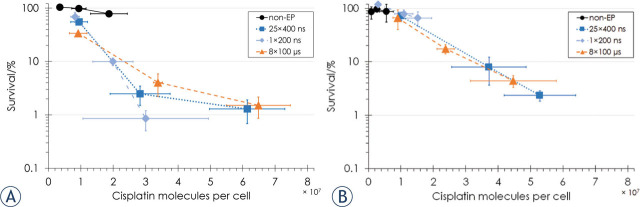
Cell survival as a function of the number of cisplatin molecules per cell for **(A)** CHO cells and **(B)** B16F1 cells in nonelectroporated (non-EP) cells (black circles) and cells electroporated with 25 × 400 ns pulses at 3.9 kV/cm, 10 Hz repetition rate (dark blue squares), 1 × 200 ns pulse at 12.6 kV/cm (light blue diamonds) or 8 × 100 μs pulses at 1.1 (CHO) or 0.9 (B16F1) kV/cm, 1 Hz pulse repetition rate (orange triangles). Bars represent standard deviation. Survival data were combined from the previous8 (for non-electroporated CHO cells and CHO cells electroporated with 25 × 400 ns and 8 × 100 μs pulses) and the present study (for B16F1 cells, additional non-electroporated CHO cells and CHO cells electroporated with 1 × 200 ns pulse).

